# Chitosan nanocarriers loaded with Egyptian *Calligonum comosum *L'Hér. Extract: an eco-friendly approach for investigating triple-action biological activities

**DOI:** 10.1186/s12906-025-05047-x

**Published:** 2025-09-24

**Authors:** Yasser I. Khedr, Soliman M. Toto, Salama M. El-Darier, Mostafa K. Hafez, Abdel-Hamid A. Sakr, Magdi A. Ali, Mohamed Zakaria El-Sayed, Aya M. Helal

**Affiliations:** 1 Department of Physics, Faculty of Science, Damanhour University, Damanhour, Egypt; 2 Botany and Microbiology Department, Faculty of Science, Alexandria University, Alexandria, Egypt; 3 Institute of Graduate Studies and Environmental Research, Damanhour University, Beheira, Egypt; 4 Medical Imaging Sciences, College of Health Sciences, Gulf Medical University, Ajman, United Arab Emirates; 5 Department of Medical Laboratory Technology, Faculty of Applied Health Sciences Technology, Pharos University in Alexandria, Alexandria, Egypt

**Keywords:** Green chemistry, Nanomedicine, Cytotoxicity, Antimicrobial nanocomposites, MIC

## Abstract

**Background:**

*Calligonum comosum* L’Hér. is a large perennial shrub that grows in the desert. This plant inhabits much of the Egyptian desert, but has not yet been scientifically validated. Herein, we demonstrate the antimicrobial, anti-inflammatory, antioxidant, and anticancer properties of the ethanolic extract of the Egyptian *C*. *comosum* (CE) and its novel eco-friendly chitosan nano-formula (CE/CsNPs), which is prepared using citrate as a crosslinker.

**Methods:**

The produced plant ethanolic extract (CE) was chemically analyzed. The eco-friendly CE/CsNPs were successfully developed through the conjugation of CE with different concentrations (200, 350, and 400 mg/100 mL) within the chitosan nanoparticle; then, it was fully characterized for the determination of the optimum formula and followed by the assessment of the antioxidant effects, proinflammatory cytokines, evaluation of cytotoxicity against different types of cancer cell lines, and estimation of antimicrobial effects of CE and CE/CsNPs.

**Results:**

The eco-friendly CE/CsNPs-2 encapsulating 350 mg of CE was selected for the in vitro studies, as it displayed NPs with a small size and a surface charge of 37.7 ± 1.91 mV with the best encapsulation efficiency (70.15 ± 1.58%) within the CsNPs, and it showed a good release profile. Transmission electron microscope (TEM) images confirmed the discrete spherical shape of CE/CsNPs-2, with no aggregations, and revealed a size range of 49.69 to 71.93 nm. FTIR displayed the successful incorporation of CE into the eco-friendly CsNPs. Regarding their biological activity, CE/CsNPs have superior efficacy with high cytotoxicity against cancer cell lines and antioxidant effects. RT-qPCR gene expression assessments displayed that CE/CsNPs resulted in a remarkable downregulation of the inflammatory cytokines’ mRNA transcript levels (*p* < 0.05). It enhanced the antimicrobial effect, with an inhibition zone of 12.34 ± 0.75 mm for *Escherichia coli*, 16.41 ± 1.01 mm for *Staphylococcus aureus*, and 18.74 ± 1.13 mm for *Candida albicans*.

**Conclusions:**

The CE possesses various biological activities, and the developed CE/CsNPs could serve as an appealing formulation to be further investigated for use in anticancer regimens.

**Graphical Abstract:**

Synthesis of eco-friendly chitosan nanoparticles loaded with ethanolic extract of *C. comosum* (CE/CsNPs) by using citrate as an organic cross-linker. The optimum CE/CsNPs formulation loaded with 350 mg/100 mL of the extract with the highest entrapment efficiency (EE)% is a promising anticancer candidate, displaying anticancer, antioxidant, and antimicrobial *in vitro* potencies.

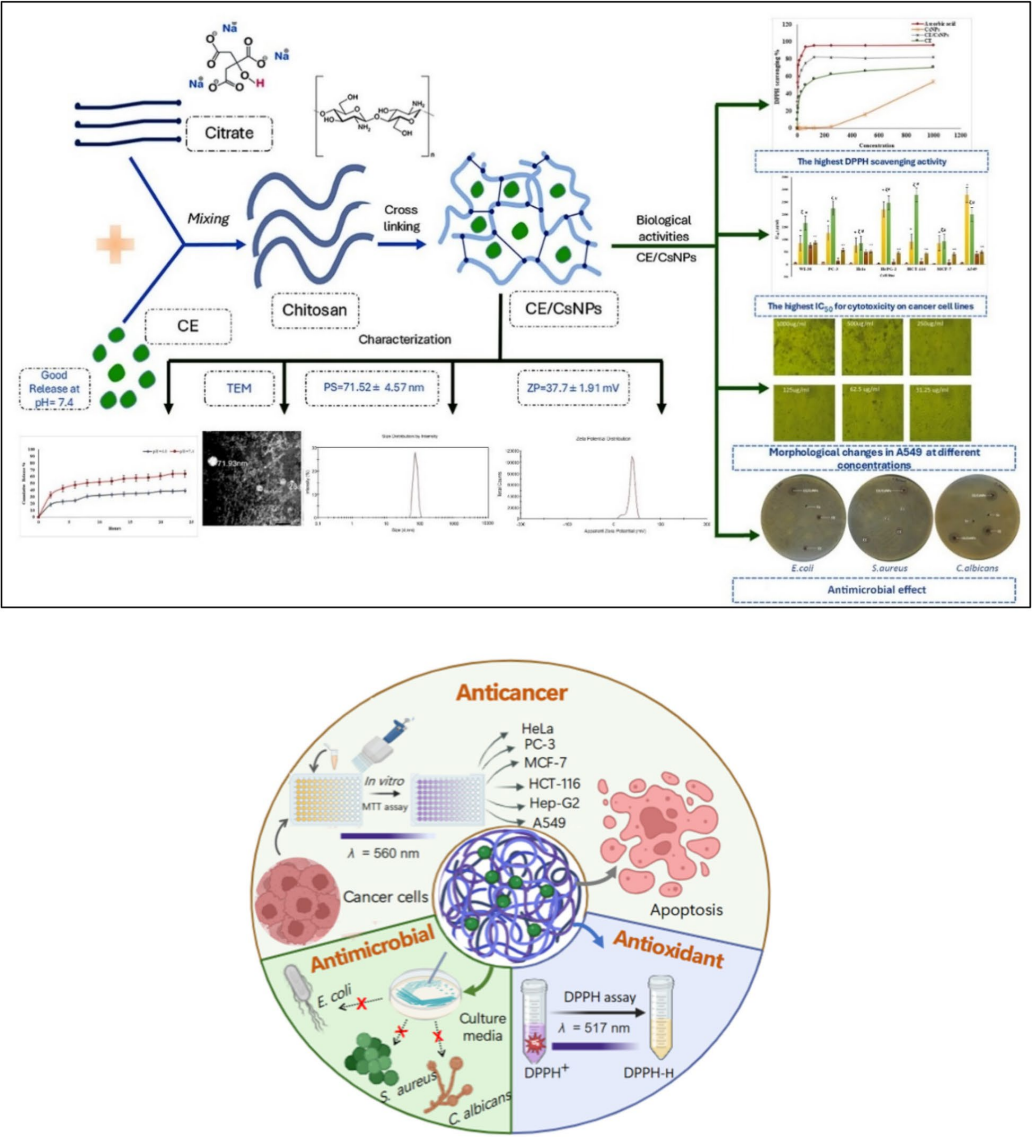

**Supplementary Information:**

The online version contains supplementary material available at 10.1186/s12906-025-05047-x.

## Introduction

Plant-based natural products, including isolated pure compounds and solvent extracts, provide incentives for drug discovery [1]. Indeed, the existence of phytochemical compounds that are generated as secondary metabolites, such as phenolics, flavonoids, tannins, alkaloids, terpenoids, and steroids, gives the plants their medicinal and therapeutic characteristics, particularly their anti-allergic, antioxidant, antibacterial, and anti-inflammatory potencies [2, 3].

*Calligonum comosum* L’Hér*.,* commonly known as"Arta". It belongs to the *Polygonaceae* family. It is a subshrub or shrub that grows primarily in the desert or dry shrubland biome. *C. comosum* is widely developing in arid and semi-arid regions, and it is particularly adapted to sandy environments. This species is distributed in North Africa, the Sahara, Socotra, West Asia, and the Arabian Peninsula [4]. In Egypt, *C. comosum* is found mainly in the Eastern and Western Deserts, the Sinai Peninsula, coastal regions bordering the Red Sea, and the Mediterranean Coastal Strip [5]. The species plays a critical ecological role, stabilizing dunes and providing shelter and food for desert wildlife. It is also noted for its remarkable tolerance to drought and salinity, making it an essential component of Egypt’s desert flora [6]. The plant has an extensive, deep root system, enabling it to access groundwater and firmly anchor in sandy soils. So far, no research has explored the potential biological activities of Egyptian *C. comosum*, which was previously studied in other regions and possesses biological components that differ geographically; hence, the biological efficacy of its extracts exhibits variability [7, 8].

Considering the biological activities of *C. comosum*, Lajter et al. (2013) [9] highlighted the remarkable anticancer characteristics of this plant through its ability to halt the cell cycle and trigger apoptosis in the triple-negative breast cancer cells, thereby providing a promising potential anticancer alternative for the treatment of malignant breast tumors. Several bioactive metabolites, including quercetin-3-O-glucuronide, catechin, kaempferol 3-O-glucoside-7-O-rhamnoside, and derivatives of pentanoic acid, are responsible for this plant's potential anticancer properties. As well, Alzahrani et al. (2021) [8] illustrated that the antioxidant and anticancer potentials of *C. comosum’s* fruit hairs against liver cancer cells are related to the potency of its phytochemical contents, including the terpenoids, alkaloids, flavonoids, and phenolics. Furthermore, *C. comosum* isolated from the United Arab Emirates (UAE) displayed impressive antibacterial potencies due to the presence of several fatty acid derivatives, which play an important role in the defense against various bacterial and fungal strains [10].

Currently, the main obstacle to effective treatments is the capability of the active compounds to pass through the cell membrane. Drugs and naturally occurring biologically active compounds possess a low bioavailability because of inadequate absorption through biological membranes and sensitivity to changes in pH [11]. Consequently, synthesizing small-sized drug-loaded nanoparticles (NPs) enables their passage across the membrane and active targeting of specific tumor cells through recognizing the expression of surface receptors [12]. For extended periods, these sustained-releasing systems ensure a regulated release of bioactive substances, hence improving their bioactivity profiles [13].

Chitosan (Cs) is considered one of the ideal biological substances for its nontoxicity, hemocompatibility, and being a biodegradable polysaccharide that readily interacts with a crosslinker under mild conditions, which makes it safer for targeted drug delivery [14]. Several drug delivery systems have been employed using chitosan nanoparticles (CsNPs) as a carrier. However, the presence of chemicals, including acetone, methanol, sodium tripolyphosphate, and glutaraldehyde as reducing agents, is an essential limitation of NPs synthesis, which increased the demand for eco-friendly methodologies for the preparation of NPs [15]. Interestingly, citrate is an organic compound that could produce polymeric networks by crosslinking more than one type of biopolymer. Citrate has been widely employed as a cross-linking agent for a diverse variety of carbohydrates, including starch, chitosan, chitin, cellulose, and pectin [16].

By considering the previous insights, the objective of our study is to investigate the biological activities of the *C. comosum* and to develop CsNPs loaded with ethanolic extract of *C. comosum* (CE) using citrate as an organic crosslinker for improving the bioavailability of the extract. The influence of different CE concentrations on the characteristics of the synthesized eco-friendly CE/CsNPs was investigated. The optimum formulation was identified by determining the EE%, PS, PDI, and ZP of the studied formulations. TEM further examined the morphology of the selected CE/CsNPs, and *in vitro* release was performed at different pH values to determine the rate and extent of active availability of the CE to the body. Until now, no research work has inspected the chitosan nanocarriers loaded with *C. comosum*. Consequently, this investigation is the first to evaluate the influences of the selected CE/CsNPs formulation in comparison to the free CE on various *in vitro* biological activities, which involved evaluating the cytotoxicity against several cancer cell lines to determine the potential of CE/CsNPs as an anticancer agent, analyzing the antimicrobial properties against bacteria and fungi, and examining their characteristics related to anti-inflammatory and antioxidant potencies.

## Materials and methods

### Materials

The cell lines in this study were acquired through a holding firm for vaccines and biological products (VACSERA), Egypt, and obtained from the American Type Culture Collection (ATCC), Manassas, VA, USA. The specific cell lines are human lung fibroblast (WI-38), human prostate cancer (PC-3), human hepatocellular carcinoma (Hep-G2), human epithelioid cervix carcinoma (HeLa), human mammary gland breast cancer (MCF-7), human colorectal carcinoma (HCT-116), and human lung cancer (A549) cell lines. RPMI-1640 medium, chitosan (Cs with a deacetylation degree of 85%, 50–190 kDa, and low molecular weight), sodium citrate, 3-(4,5-dimethylthiazol-2-yl)−2−5-diphenyltetrazolium bromide (MTT), doxorubicin, penicillin, streptomycin, dimethyl sulfoxide (DMSO), 2,2-diphenyl-1-picryl-hydrazyl (DPPH), and phosphate-buffered saline (PBS) were supplied from Sigma Aldrich, USA. The RIPA lysis buffer was provided by Thermo Fisher Scientific, USA. Fetal bovine serum was donated by GIBCO, UK. SYBR green master mix, reverse transcriptase, and nuclease-free H₂O were provided by Bio-Rad Laboratories (California, US). Antibiotic disks and culture media were obtained from Lab Express, USA. Acetic acid glacial (Grade AR, CH₃COOH, 60.05 g/mol) and ethanol were purchased from El-Nasr Pharmaceutical Chemicals Co. (Cairo, Egypt).

### Methods

#### Collection of *C. comosum* and preparation of its ethanolic extract (CE)

*Calligonum comosum* (Fig. S1) shoots were collected in November 2022 from Dakahlia Governorate, Egypt, 31°26'33.2"N 31°28'54.0"E. Plant material was gathered under the relevant national regulations and the guidelines set by the International Union for Conservation of Nature (IUCN) [17] and the Convention on the Trade in Endangered Species of Wild Fauna and Flora (CITES) [18]. Permission to collect the plant specimens of the species under investigation for scientific purposes was obtained from the Department of Botany and Microbiology at the Faculty of Science, Alexandria University.

The plant was identified by Soliman M. Toto, Associate Professor of Flora and Ecology, Department of Botany and Microbiology, Faculty of Science, Alexandria University. A voucher specimen was deposited in the Herbarium of Alexandria University (ALEX) at the Faculty of Science, deposition number 4115.

**Fig. 1:**
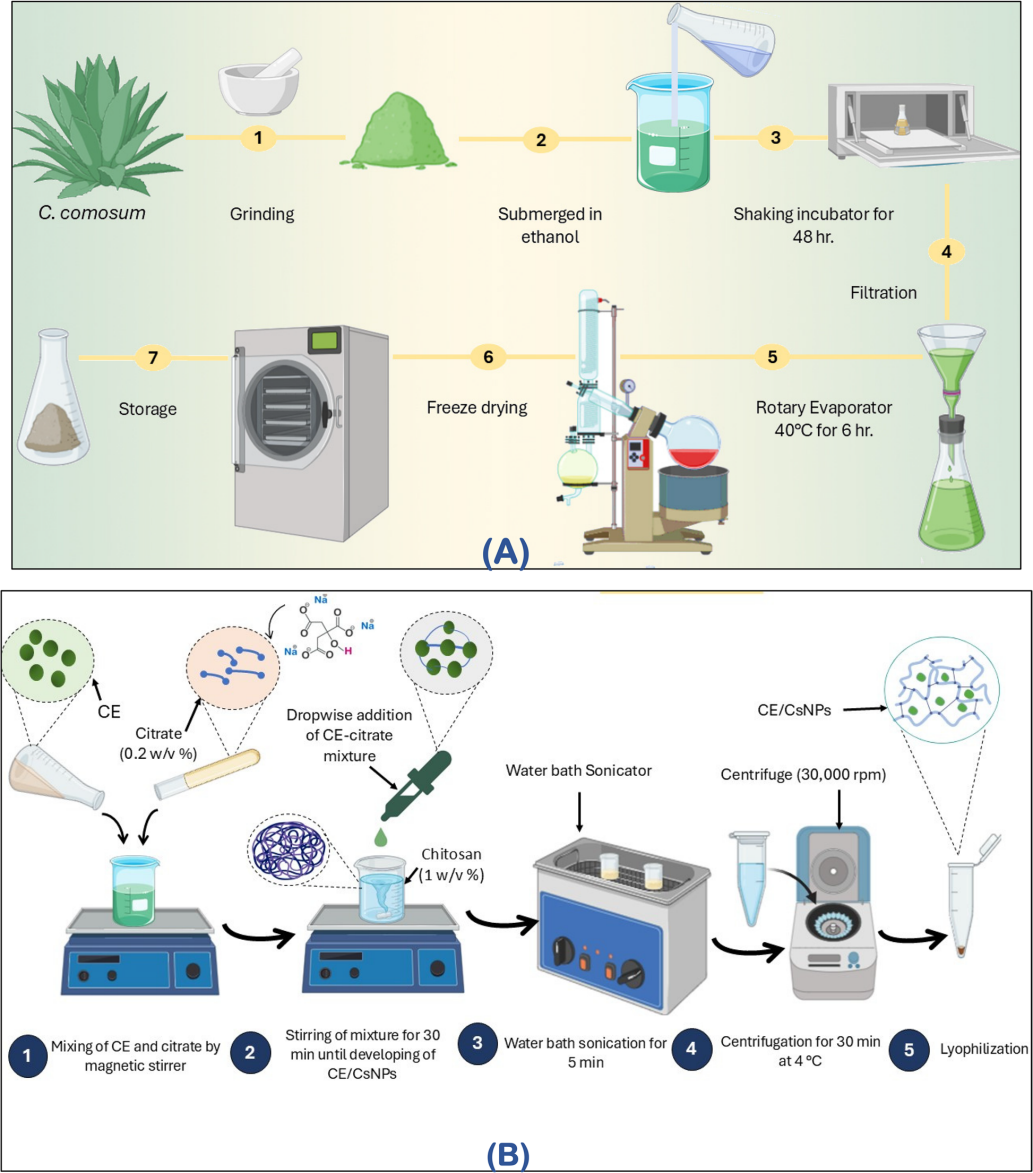
Scheme diagram showing the preparation steps of (**A**) Ethanolic extract of *C. comosum* (CE) and (**B**) CE chitosan nanoparticles (CE/CsNPs)

For this investigation, 5 kg of fresh *C. comosum* was collected. Following an oven drying procedure at 60 °C, the plant was weighed, crushed, and fully immersed in 2 L of 70% (v/v) ethanol for each 200 g of dry weight. The mixtures were maintained for 48 hr in a shaking incubator at room temperature and then filtered. For complete recovery of the ethanol from the extract, the filtrate was dried for 6 hr in a rotary evaporator, which was set at 40°C. The resultant ethanol extract was lyophilized using a freeze-dryer lyophilizer to achieve complete dryness, yielding a 68 g powder that was stored in the refrigerator till being used (Fig. 1A).

### Analysis of CE

#### Determination of bioactive components by GC/MS

The bioactive components in the *C. comosum* ethanolic extract were determined using a direct capillary column TG-5MS and a trace GC1300-ISQ mass spectrometer. The temperature of the column oven was set at 60 °C, and increased by 5 °C per minute until it reached 200°C. Helium was employed as the carrier gas. The sample was diluted and then automatically injected by the Autosampler AS1300 and GC in split mode. The components were identified via comparison of their retention periods and mass spectra to previously recognized mass spectral databases [19].

### Preparation of CE/CsNPs formulations

For the preparation of the Cs solution, low molecular weight Cs (0.2%) was mixed with deionized water, and constant stirring was applied at 1200 rpm for 1 hr at 37 °C until the solution was completely dissolved. Upon adding 1% w/v of acidified water to the deacetylated Chitosan, the mixture was then sonicated till it turned clear. As for the cross-linker, 0.2% w/v of citrate was dissolved in deionized water, added dropwise into the Cs solution with continuous stirring for 30 min at 500 rpm, and the CsNPs were produced spontaneously [20].

To determine the ideal CE concentration to be loaded in the CsNPs, filtered ethanolic extract of *C. comosum* at various concentrations of 200, 350, or 400 mg/100 mL was mixed thoroughly with the sodium citrate solution (0.2% w/v). Thereafter, the drug solution was added in a dropwise manner to 25 mL of the Cs solution (0.2%) and further magnetically stirred at room temperature for 30 min to achieve the CE/CsNPs formulations. The resulting NPs were ultrasonicated at the maximum power (130 kW) to reach a consistent particle size; afterwards, ultracentrifugation (30,000 rpm) at a temperature of 4 °C for 30 min was applied to separate the developed formulation from the suspension. Finally, the resulting pellets were then washed and lyophilized (Novalyphe-NL 500 freeze-dryer; Savant Instruments, NY, USA) at −90°C for 24 hr (Fig. 1B).

### Characterization of CsNPs and CE/CsNPs

#### Size analysis and zeta potential of synthesized NPs

The Nano-Zeta sizer (Malvern and Worcestershire, UK) measured the particle size (PS), zeta potential (ZP), and polydispersity index (PDI) of the synthesized different CE/CsNPs formulations by using the dynamic light scattering (DLS) technique [21]. This technique depends on the changes in light diffraction generated by the Brownian motion of synthesized NPs in the dispersions. Before measuring the parameters, dilution of the dispersions was applied, and then the measurements were done in triplicate to estimate the mean of the responses.

### Entrapment efficiency (EE%) and loading capacity (LC%)

The CE’s definite amount loaded into the different formulations is identified as the entrapment efficiency (EE)%. Ultracentrifugation at 11,000 rpm of the freshly synthesized CE/CsNPs formulation in a predetermined amount for 35 min at 4 °C is used. The obtained supernatant was then filtered and subjected to a UV/Vis spectrophotometer (Helios Alpha, Unicam) at a maximum wavelength (λ) of 210 nm. Equations (1) and (2) are applied to calculate the EE% and LC%, respectively [22].

1 $$EE\%= \frac{Initial\,amount\,of\,CE(mg)-Free\,CE(mg)}{Initial\,amount\,of\,CE(mg)}\text{x}100$$

2 $$LC\% = \frac{Initial\,amount\,of\,CE(mg)-Free\,CE(mg)}{Mass\,of\,the\,carrier\,(CsNPs)(mg)}\text{x}100$$

#### Transmission electron microscopy (TEM)

The morphological size and shape of the optimal CE/CsNPs formulation and drug-free CsNPs were determined using TEM (Joel JEM 1230, Tokyo, Japan) at an acceleration voltage of 80 kV [23].

#### Assessment of drug release

The dialysis bag technique (VISKING^®^ 28 mm, MWCO 12,000–14,000; Serva, Heidelberg, Germany) was applied to estimate the active compounds of CE encapsulated in the CE/CsNPs-2 formula. At 37 °C, the bag was dispersed in a release medium containing buffer with either pH 4.0 or pH 7.4. Then the complete system is settled on the shaker (WiseCubeVR WIS Precise Shaking Incubator, Wertheim, Germany).

To keep the intended conditions, at specified intervals, 2 mL aliquots were obtained and replaced with an equivalent volume of the freshly prepared buffer. The collected aliquots were then centrifuged at 4 °C at 11,000 rpm for 30 min. UV spectroscopy (Helios Alpha, Unicam) was applied for the determination of CE content in the supernatant, and absorbance was recorded (maximum λ of 280 nm). The trials were performed in triplicate, and then the exact concentration of the extract’s released compounds was measured according to its calibration curve estimated from predetermined amounts of CE under the same conditions and calculated according to Eq. 3 [11].

3 $$CE\;release \left(\%\right)=\frac{CE\;released\;at\;definite\;time}{Total\;amount\;of\;CE\;entrapped\;within\;nanoparticle}\text{x}100$$

### FT‑IR analysis

Previously lyophilized samples were used to measure their infrared spectra with the aid of Shimadzu 8400S FT-IR spectroscopy, Japan. The chemical structures of Cs, CE, CsNPs, and CE/CsNPs lyophilized samples were studied to identify the distinctive peaks that correspond to their characteristic functional groups. FT-IR spectra were recorded at room temperature with λ ranging from 4000 cm⁻^1^ to 400 cm⁻^1^ [24].

### Evaluation of in vitro antioxidant capacity of CE/CsNPs

The procedure outlined by Shetta et al. (2019) [25] was employed with a few alterations to examine the free radical scavenging capacity of CE, CsNPs, and CE/CsNPs. Briefly, 0.1 mM of the DPPH stock solution was dissolved in ethanol. Subsequently, 1 mL was added to 3.5 mL of various serially diluted amounts of the samples (1000, 500, 250, 125, 62.5, 31.25, 15.62, 7.81, and 3.9 µg/mL). Ascorbic acid was used as a standard. The samples were then agitated and incubated in the dark for 30 min to react with ethanolic DPPH. The changes in absorbance were estimated at λ=517 nm using a UV/Vis spectrophotometer (Shimadzu, model UV-1601 PC, Kyoto, Japan). These measurements were done in triplicate, and the inhibition percentage was calculated for each sample according to Equation (4). The linear regression analysis is used to calculate the IC_50_ values to indicate the antioxidant capacity.

4 $$DPPH\,activity\,(\%)=\frac{Absorbance\,(Control)-Absorbance\,(Sample)}{Absorbance\,(Control)}\text{x}100$$

### Determination of the CE and CE/CsNPs cytotoxic activities

The MTT assay was conducted to evaluate the tested compounds’ inhibitory impact on the proliferation of WI-38, PC-3, HeLa, Hep-G2, HCT-116, MCF-7, and A549 cell lines. This colorimetric test relies on mitochondrial succinate dehydrogenase in living cells, converting yellow MTT into a purple formazan derivative. RPMI-1640 medium containing 10% fetal bovine serum was utilized for culturing the cells. As a standard anticancer drug, doxorubicin was used.

The cells were incubated in the media at a density of 1.0x10^4^ cells/well for 48 hr in a 96-well plate at 37^◦^C, and a 5% CO_2_ condition was maintained. At the end of the incubation period, the cells were treated with graded concentrations of Cs, CE, or CE/CsNPs (31.25, 62.5, 125, 250, 500, and 1000 μg/mL) and re-incubated for 24 hr. Afterward, the media were aspirated, and 20 µL of MTT solution (5 mg/mL) prepared in PBS was then added, followed by incubation for 4 hr. DMSO was added at a volume of 100 µL to each well to dissolve the purple formazan. The absorbance of each sample was measured at 560 nm using a plate reader (EXL 800, USA). The percentage of relative cell viability was calculated as [(ODt/ODc)] × 100%, where ODt represents the mean optical density of cells treated with the different treatments, and ODc represents the mean optical density of untreated ones. The 50% inhibitory concentration (IC_50_), which represents the concentration at which cell viability was reduced by 50%, was calculated using GraphPad Prism software (San Diego, CA, USA). This was achieved by generating and analyzing dose-response curves from the experimental data [26].

### RNA extraction and RT-qPCR analysis of inflammatory cytokines

The gene expression of cytokines in A549 and Hep-G2 cells treated with CE/CsNPs, CE, or CsNPs was estimated using real-time PCR with SYBR Green and compared to their expression in untreated cells. The seeded cells were exposed to the investigated compounds at concentrations equivalent to their IC_50_ values for 48 hr [24]. Subsequently, the lysis of cells was performed by using the RIPA buffer containing phosphatase and protease inhibitors. The cell lysate was centrifuged at 15,000 g for 10 min at 4°C. Thereafter, RNA extraction was done by RNeasy Mini Kit (Qiagen Inc., CA, USA), following the manufacturer's protocol. Thermo Scientific NanoDrop™1000 Spectrophotometer and its analytical software (V3.7, DE, USA) were used to estimate the RNA concentration and purity at A260 and A280 nm. Then, the cDNA synthesis was conducted according to the manufacturer’s instructions of the iScript One-Step RT-PCR Kit with SYBR Green (BIO-RAD). Specific primers for IL-1β, TNF-α, and β-actin were acquired from Bioneer Corp., Korea, and employed (Table S1). The 2^−ΔΔCt^ method was used to calculate the target genes'fold change compared to β-actin as a housekeeping gene to ascertain the relative mRNA expression level for a particular gene [27].

### Evaluation of the CE and CE/CsNPs antimicrobial potencies

The disc diffusion assay was used to determine the antimicrobial activities of the tested compounds against gram-positive bacteria (*Staphylococcus aureus,* ATCC 6538) and gram-negative bacteria (*Escherichia coli,* ATCC 8739), as well as the determination of their antifungal activity on *Candida albicans* (ATCC 10221). The microbes were obtained from the routine Microbiology laboratory of Alexandria Main University Hospital. Pure cultures of each strain were subcultured on Mueller-Hinton agar (MHA) medium and cultivated at 37 °C for 24 hr at pH 7.2–7.4 [28]. The actively growing suspensions were adjusted to match the turbidity of a 0.5 McFarland standard (1.5 × 10^6^ CFU/mL). The test plates were cultivated with each tested inoculum, which was spread using a sterile cotton swab to ensure equal seedling distribution over the plates. The prepared CsNPs, CE, or CE/CsNPs were dissolved in DMSO at a concentration of 1 mg/mL. The disc diffusion assay was performed according to Alghamdi et al. (2023) [29]. After applying the filtration method for sterilization, 50 µL of each treatment was used to soak the sterilized discs (6 mm diameter), which were placed in the medium. As for positive controls, ampicillin (AMP, 30 mcg/disc) for bacterial strains and clotrimazole (CC, 10 mcg/disc) for fungi were employed (HIMEDIA, India). DMSO (0.5% v/v) and blank (media) samples were applied as negative controls. The plates were left at room temperature for 1 hr to allow diffusion of the tested samples, then incubated at 37 °C for 24 hr. Each experiment was done in triplicate, and the inhibition zones were measured in millimeters (mm); then the percentage of activity index for each treatment was calculated according to Equation (5)[30].

5 $$Activity\,Index\,(\%)= \frac{Zone\,of\,inhibition\,(mm)\,by\,tested\,compound\,(diametre)}{Zone\,of\,inhibition\,(mm)\,by\,control\left(diametre\right)}x100$$

#### Determination of minimum inhibitory concentration (MIC), minimum bactericidal concentration (MBC), and minimum fungicidal concentration (MFC)

The MIC and MBC of CE and biosynthesized CE/CsNPs were determined by the broth microdilution method in Mueller–Hinton (MH) broth in 96-well plates using DMSO with a serial double dilution of CE, CE/CsNPs, or CsNPs over the range of 1.95–1000 μg/mL, as reported by Javan Bakht Dalir et al*.* (2020) [31] with some modifications. A stock solution of CE, CE/CsNPs, or CsNPs was prepared by mixing 10 mg of the used treatment with 10 mL of DMSO (1000 μg/mL). Briefly, 95 μL of the nutrient broth and the studied microorganism were dispensed into each well. Then, 100 μL of CsNPs, CE, or CE/CsNPs stock solution (1000 μg/mL) was added into the first well. Afterwards, 7 successive wells were then filled with 100 μL of their serial dilutions. The last well contains 195 μL of the nutrient broth without any compound. A negative control (broth containing DMSO) and a positive control (broth and inoculum) were used. The plate is placed on a shaker at 200 rpm for proper mixing and then incubated at 35 ± 2 °C for 24 hr. The MIC endpoint is the minimum concentration of CE or CE/CsNPs, which entirely inhibits the tested microorganism’s growth in the microdilution wells. The turbidity was evaluated by estimating the OD at 630 nm by using a BioTek 800 TS microplate reader, and each test was performed in triplicate [30].

For the estimation of MBC or MFC, which is the minimum concentration of the CE, CE/CsNPs, or CsNPs required to destroy the final inoculum by about 99.9% for MBC [32] and 98% – 99.9% for MFC [33]. After determining the MIC of CE, CE/CsNPs, or CsNPs, 20 μL from each well that showed no microbial growth was seeded on MHA medium and then kept at 37 °C for 24 hr to determine the viable organisms [30].

### Statistical analysis

The obtained data were statistically analyzed using SPSS version 20.0 (SPSS, IBM, USA) by one-way analysis of variance (one-way ANOVA), and then post-hoc comparison between groups was applied. The value of *P* < 0.05 was used as a limitation for the results'significance. The results in this investigation are expressed as mean ± standard deviation.

## Results and discussion

### Analysis of CE by GC/MS

The GC–MS is one of the best techniques for determining the presence of gaseous components, alcohols, acids, phenolic compounds, and esters [34]. The analysis of CE showed 25 peaks with 54 active chemicals (Fig. S2), with Linoleic acid esters being the most prevalent at 25.04%; followed by 6-octadecenoic acid and octadec-9-enoic acid with 21.08%; decanedioic acid, dibutyl ester, sebacic acid, butyl ethyl ester and hexadecanoic acid ethyl ester (7.71%); n-hexadecanoic acid, methyl (tetramethylene) silyl ester, α-D-galactopyranoside methyl-4-O-decyl- (4.37%); fluocinonide, cholestan-3-one, cyclic 1,2-ethanediyl acetal, (5α)- and D-glucopyranose (3.95%); geldaramycin, cyclopropaneoctanoic acid, 2-octyl-, cis-methyl ester and E,E,Z-1,3,12-nonadecatriene-5,14-diol (2.97%); ethyl 14-methyl-hexadecanoate and α-D-mannopyranose,1-O-(trimethylsilyl)-,2,3:4,6-dibutaneboronate (2.94%); methyl 5,13-docosadienoate butyl 9,12-octadecadienoate and n-propyl 9,12-octadecadienoate (2.85%); hexadecanoic acid, methyl ester and nonanedioic acid, bis (2-methylpropyl) ester (azelaic acid) (2.32%); 4-[5-(4-Chloro-2-cyclohexyl-phenoxymethyl)- 1,2,4 oxadiazol-3-yl]- furazan-3-ylamine and stigmasta −5,22-dien-3-ol, acetate, 4,4-dimethyl-5α-cholest-7-en-3β-ol with an area of 2.18%; aspidofractinine-1-carboxaldehyde, 17-methoxy-3-oxo-, (2à,5à), rhoifolin, and pregna-4,6-diene-3,20-dione, 17-hydroxy-6,16 α-dimethyl- (1.99%).

Collectively, these findings showed the existence of phytosterols, alkaloids, polyphenols, essential oils, and carbohydrates. All of these compounds are bioactive and have various antibacterial, anticancer, anti-inflammatory, and antioxidant capabilities, as presented in Table 1. According to reports by Soliman et al. (2021) [7] and Riadh et al. (2011) [35], GC–MS analysis of *C. comosum* isolated from the UAE and Tunisia displayed the presence of common organic compounds, including 9,12-octadecadienoic acid (linoleic acid), 6-octadecenoic acid, pentadecanoic acid, and hexadecanoic acid ethyl ester. Unsurprisingly, the Egyptian plant possessed other biological compounds that were not found in *C. comosum* collected from other regions. This could be explained by the fact that different climatic zones can have a substantial impact on the chemistry and activities of medicinal plants with the same phenotype.

**Table 1:** The major compounds identified in the ethanolic extract of *C. comosum *(CE), retention times (RT), percentage compositions, chemical structures, molecular formulas, molecular weights (Mwt), and their biological activities

**Table 2:** Colloidal properties of chitosan nanoparticles formulations loaded with different concentrations of ethanolic extract of *C. comosum* (CE/CsNPs) and chitosan nanoparticles (CsNPs); showing their measured particle size, polydispersity Index (PDI), Zeta potential (ZP), entrapment efficiency % (EE) and loading capacity (%) data (*n* = 3)

Nano formulation	Concentration of the Extract (mg/100 mL)	Particle size (nm)	PDI	ZP (mV)	EE%	LC%
CsNPs	–	42.54 ± 3.65	0.29 ± 0.03	69.2 ± 2.37	–	–
CE/CsNPs-1	200	60.57 ± 3.25	0.24 ± 0.01	40.1 ± 2.56	60.84 ± 2.97	17.58 ± 1.95
CE/CsNPs-2	350	71.52 ± 4.57	0.35 ± 0.04	37.7 ± 1.91	70.15 ± 1.58	20.47 ± 2.14
CE/CsNPs-3	400	130.5 ± 1.02	0.63 ± 0.12	24.5 ± 2.45	55.75 ± 3.12	15.78 ± 1.48

### Synthesis and characterization of synthesized CE/CsNPs formulations

#### PS, PDI, ZP, and EE% measurements

The bioactive compounds in plant extracts, such as alkaloids, flavonoids, and terpenoids, are highly soluble in water but have limited bioavailability and efficacy due to their large molecular sizes, poor absorption, and inability to pass through lipid membranes [76]. Nanotechnological approaches have overcome these challenges, allowing the use of compounds with different properties in the same composition [77]. Furthermore, the latest studies are focusing on finding sustainable, eco-friendly, and creative techniques to synthesize nanomaterials that are less hazardous to the environment and lifeforms [78, 79].

This study focused on developing eco-friendly CsNPs using citrate as a cross-linking agent, loaded with the optimum concentration of CE. Citrate has been used as a sustainable cross-linker for bio-polymeric materials due to its affordability and being naturally present. It is also known for its tensile characteristics and antimicrobial capabilities through its cross-linking effects [80].

**Fig. 2:**
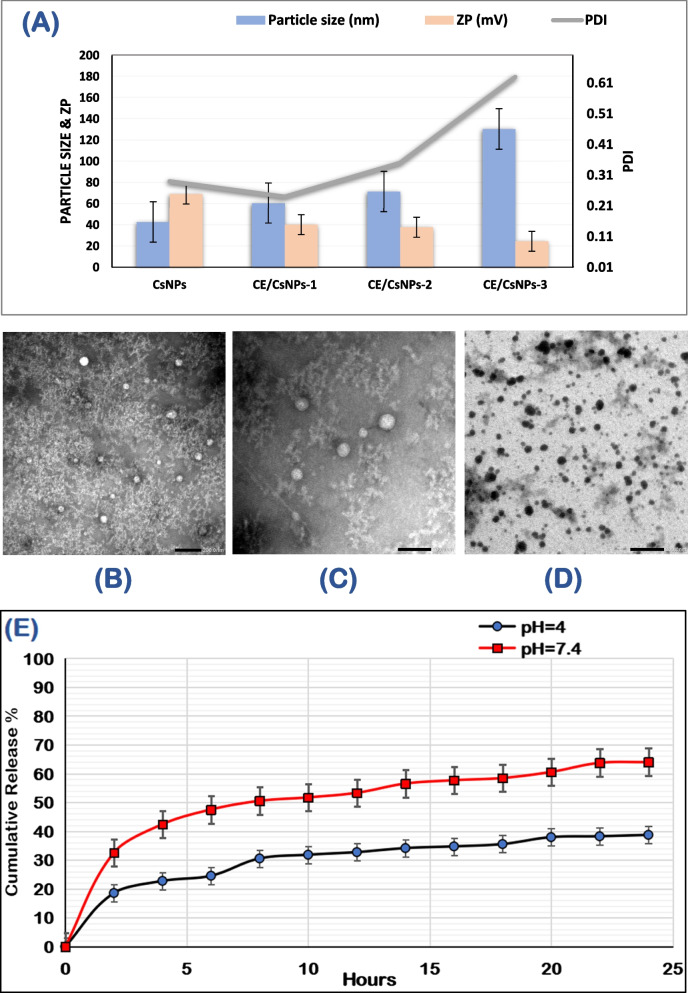
**A** Colloidal properties of different chitosan nanoparticles formulations, **B**, **C** morphology examination of the synthesized CE-loaded chitosan nanoparticles (CE/CsNPs), **D** chitosan nanoparticles (CsNPs) by TEM, and (**E**) in vitro release of ethanolic extract of *C. comosum* (CE) compounds from chitosan nanoparticles (CsNPs) at pH 7.4 and pH 4.0. PDI: Polydispersity Index; ZP: Zeta potential

As illustrated in Table 2, the synthesized CsNPs have a PS of 42.54 ± 3.65 nm, a uniform size distribution with a PDI of 0.29 ± 0.03, and a ZP value of 69.2 ± 2.37 mV. These results align with previous studies showing NPs with a high positive charge due to the electrostatic repulsion between chitosan’s amino groups within the molecule [81]. A recent investigation conducted by Hadkar and Selvaraj (2025) [82] suggested that an electrokinetic potential over 30 mV is necessary to maintain stable colloidal characteristics in aqueous solutions with moderate ionic strength. Loading CsNPs with CE by different concentrations (CE/CsNPs 1–3) resulted in significant changes in colloidal properties, with an increase in PS ranging from 60.57 ± 3.25 nm to 130.5 ± 1.02 nm (Fig. 2A) due to the loading of the extract in CsNPs, as explained by Panwar et al. (2016) [83] and Nallamuthu et al*.* (2015) [84]. The increase in the PDI value, ranging from 0.24 ± 0.01 to 0.63 ± 0.12, indicates a decrease in the homogeneity in aqueous solution [24] by increasing the concentration of the extract loaded within the CsNPs. The coupling of CE compounds in the developed CE/CsNPs formulations reduced the surface charge (40.1 ± 2.56 mV to 24.5 ± 2.45 mV), possibly due to the neutralization of the positive charges of the chitosan's amino groups [85].

Interestingly, the estimated EE% values for the CE/CsNPs (1–3) formulations were 60.84 ± 2.97%, 70.15 ± 1.58%, and 55.75 ± 3.12%, respectively, validating their stability and suitability for anticancer and antioxidant applications [86]. According to the obtained data, the CE/CsNPs-2 formula possessed the highest entrapment efficiency (70.15 ± 1.58%). It displayed good particle size (71.52 ± 4.57 nm), ZP (37.7 ± 1.91 mV), and PDI (0.35 ± 0.04) among all the studied formulations; thus, it was selected as the best achieved CE/CsNPs formula for further investigation in the current research.

#### TEM analysis

TEM is an image-guided method that confirms the shape and estimated size of the selected formula (CE/CsNPs-2), calculated by the DLS [87]. Figures 2B-D shows the TEM micrographs of the developed CE/CsNPs (Fig. 2B & C) and CsNPs (Fig. 2D). Interestingly, CsNPs showed spherical NPs with a size range of 34.94 nm to 43.64 nm. As for the CE/CsNPs, the TEM image showed NPs with a spherical shape and a PS range of 49.69 nm to 71.93 nm, which is closely related to the Zeta-sizer values. This suggests that CE/CsNPs do not aggregate in aqueous media and have a narrow size distribution, allowing them to disperse uniformly throughout cancerous tissue [24, 88].

#### CE/CsNPs in vitro drug release

The percentage of cumulative drug concentration over time under various pH circumstances (pH 4.0 and pH 7.4) is displayed in Fig. 2E. Both acidic (pH 4.0) and alkaline (pH 7.4) conditions provoked a burst release of the CE. Afterwards, the release efficiency started to slow down in acidic medium (pH 4.0) with a maximum efficiency of 38% after 24 hr of dialysis. Nevertheless, the release of CE was obvious at pH 7.4, with an efficient release of 64% after 24 hr. This aligns with previous experimental studies, where CE/CsNPs-2 displayed a higher drug delivery efficiency after 24 hr in the physiological environment compared to the acidic environment [88, 89].

The behavior of CE release depends on the charging properties and how the Cs and citrate molecules interact with each other. At pH 4.0, the degree of protonation of the chitosan's amino groups is less affected, and the citrate molecules are suitably deprotonated, resulting in a strong connection between the two molecules'charges and thus preventing the release of CE compounds [90]. While raising the pH, which makes the citrate much more charged, it also neutralizes the charge density in the Cs, which causes the collapse of the NPs and the drug release. In Light of these results, the CsNPs showed a maximum release of CE at pH 7.4, which is comparable to intestinal pH. Thus, the CE/CsNPs can act effectively as an oral delivery agent, besides overcoming the stomach's acidic medium with significant delivery at physiological pH.

### FTIR analysis

**Fig. 3:**
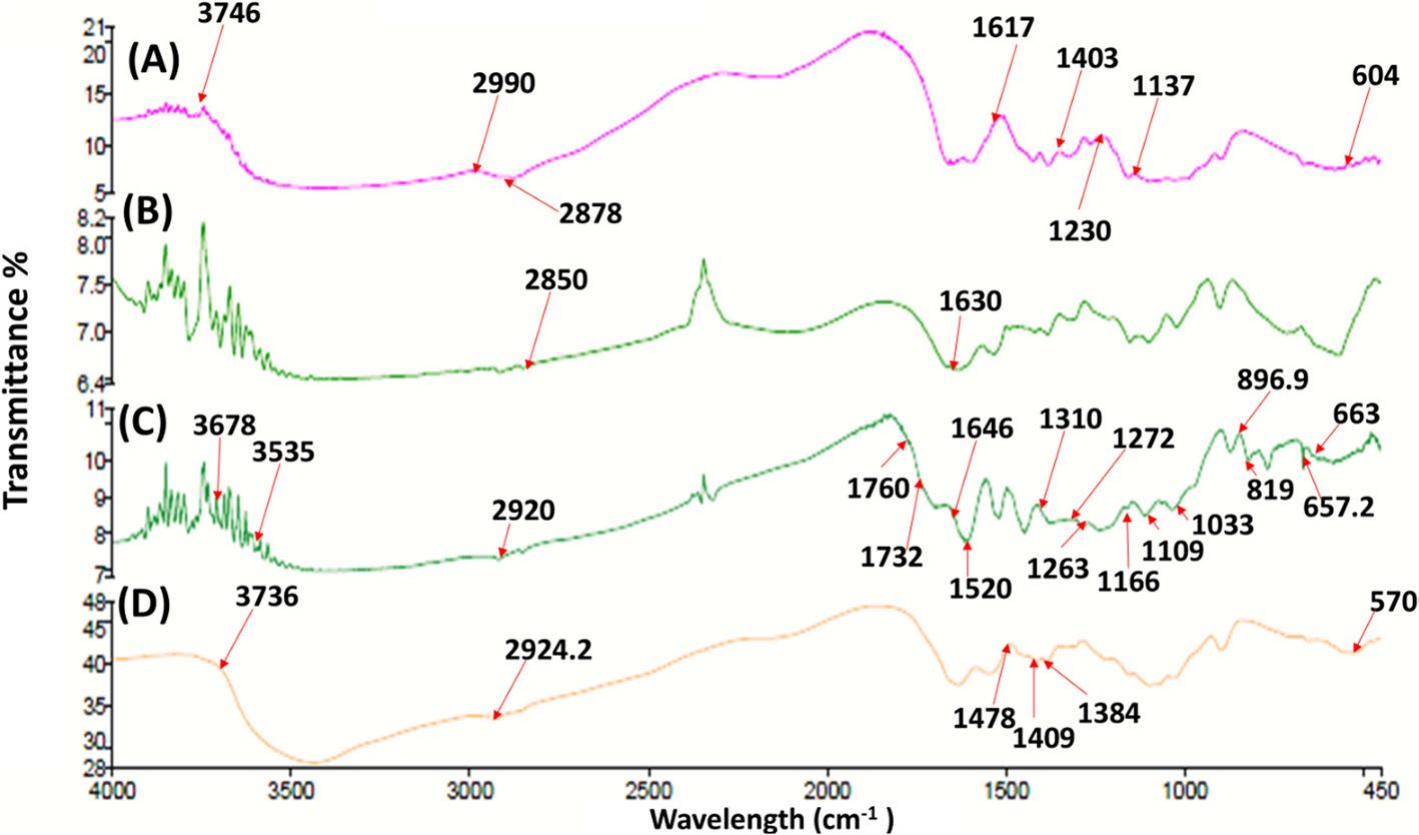
FTIR analyses for (**A**) chitosan powder (Cs), **B** chitosan nanoparticles (CsNPs), **C** ethanolic extract of *C. comosum* (CE), and (**D**) CE chitosan nanoparticles (CE/CsNPs)

The spectrum of Cs (Fig. 3A) illustrated a characteristic peak of 2990 cm^−1^ representing the vibration of NH_2_, 3746 cm^−1^ for the stretching vibration of OH, and 2878 cm^−1^ representing the C–H bond in alkanes and carboxylic groups [91], and the C = O bond in the amide I molecules could be recognized at 1617 cm^−1^ [92]. The band shown at 1403 cm^−1^ is general for OH groups bending, 1230 cm^−1^ and 1137 cm^−1^ correspond to the stretching vibrations of C–O–C bonds, and the band at 604 cm^−1^ corresponds to the pyranoside rings, which are also indicated in the previously published results by Dalal et al. (2021) [93]. Accordingly, the cross-linking of Cs polymer with citrate is expected to shift the peaks related to amide groups. Thus, comparing FTIR spectra of Cs and CsNPs (Fig. 3A & B) illustrated that the peak at 2878 cm^−1^ of the –NH_2_ groups in Cs was shifted to 2850 cm^−1^ due to the acetylation of Chitosan and cross-linking with citrate molecules. In addition, the peak at 2990 cm^−1^ related to the C–H bond in alkanes and carboxylic groups was absent in CsNPs, indicating a change in chitosan structure upon nano-formulation [16, 20]. The existence of C–O–C and NH_3_^+^, respectively, was validated by the presence of two distinctive strong bands at 1020 cm^−1^ and 1159 cm^−1^, respectively, due to chitosan cross-linking with citrate during synthesis [94].

Furthermore, a distinct peak emerged at 1382 cm^−1^ (C − O bond) that could indicate that the protonated amine group of chitosan (− NH_3_^+^) interacted with the carboxyl group of the citrate (COO^−^) [16]. The absorption bands of CE functional groups are defined in Fig. 3C. The peak that appeared at 1760 cm^−1^ is assigned to the C = O group. The bands 3678 cm^−1^ and 3535 cm^−1^ are ascribed to O–H bands, which can be found in sebacic acid, hexadecanoic acid, and octadecenoic acid, along with the presence of potent phenolic compounds represented in rhoifolin [95], which has been confirmed in the previous GC–MS results. The band that appeared at 2850 cm^−1^ belongs to the N–H bond, possibly found in geldanamycin [96]. The 2920 cm^−1^ and 1646 cm^−1^ peaks were attributed to C–H and C = O stretching, respectively [97]. The relatively small bands that were visible at 1310 cm^−1^, 1267 cm^−1^, 1166 cm^−1^, and 1109 cm^−1^ are assigned to various types of esters that are present in CE. The peak at 1033 cm^−1^ is assigned to C-F, which is possibly present in fluocinonide. Furthermore, the bands 1520.4 cm^−1^ and 657.2 cm^−1^ are attributed to the N–O and C–Cl bonds, respectively, that perhaps exist in the milbemycin B, as confirmed in the GC–MS results. The peak at 663 cm^−1^ corresponds to the C = C group. Moreover, the peak at 666.7 cm^−1^ could be ascribed to the absorption band of the C–H bond bending in the benzene ring out of plane [98].

On the contrary, the CE/CsNPs spectrum shown in Fig. 3D displayed that the characteristic peaks of CE were absent. Since the O–H peak (3736.6 cm^−1^) becomes broader, it confirms that the reaction between CsNPs and CE enhances the bonding action [99]. In addition, a shift in the C–H stretching bands at 2924.2 cm^−1^, 1478 cm^−1^, and 1384 cm^−1^, along with the disappearance of the bands at 1760 cm^−1^, 1310 cm^−1^, 869 cm^−1^, and 819 cm^−1^, which reflect the encapsulation of CE within the CsNPs. Strong bands were observed at 1635 cm^−1^ and 1546 cm^−1^ for the amide C = O stretching [100].

### CE and CE/CsNPs antioxidant activity

**Fig. 4:**
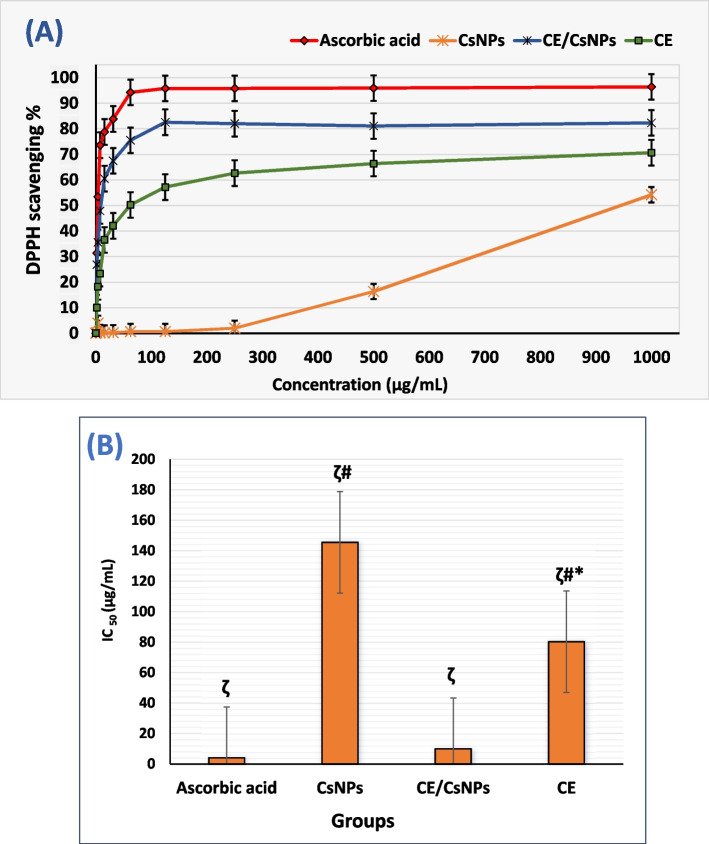
**A** The in vitro antioxidant capacity of CsNPs, CE/CsNPs, and CE; **B** Half-maximal inhibitory concentration (IC_50_) values of DPPH radical scavenging activity on CsNPs, CE/CsNPs, and CE solutions (ζ *P* < 0.0001). * *p* < 0.05 CE group *vs.* CE/CsNPs group, ^#^ *p* < 0.05 CE or CsNPs groups *vs.* ascorbic acid. In all panels, *n* = 3, results are expressed as mean ± standard deviation. CE/CsNPs: Ethanolic extract of *C. comosum* loaded in chitosan nanoparticles, CE: Ethanolic extract of *C. comosum*, CsNPs: Chitosan nanoparticles, and DPPH: 2,2-diphenyl-1-picryl-hydrazyl

The antioxidant assay was conducted on CE, CsNPs, and CE/CsNPs to evaluate their ability to scavenge free radicals. DPPH is a stable free radical with a visible deep purple color that becomes decolorized. This occurs due to the transfer of a hydrogen atom donated by the antioxidant molecules to the proper hydrazine, which reduces the odd electrons of the N-atom in the DPPH [101]. CE/CsNPs showed the highest DPPH scavenging activity, ranging from 82.3% to 26.85%, followed by CE that exhibited reducing power ranging from 70.67% to 10.02%, however the CsNPs displayed limited antioxidant activity (Fig. 4A). Notably, the reduction capacity of each tested compound was apparent with the increase in the concentration of applied treatment [102]. Indeed, the antioxidant power of *C. comosum* could be ascribed to the presence of various antioxidant biological compounds, including unsaturated fatty acids, flavones, quinones, and alkaloids (Table 1).

Previous studies showed promising antioxidant results for these compounds, including β-carboline alkaloids (peak 21) [66]. Rhoifolin, which belongs to flavones, a subclass of flavonoids (peak 25) [95], as well as aspidofractinine (peak 25) [73], and various fatty acids and fatty acid esters [37–39, 42, 47]. Additionally, it was demonstrated that β-carotenoic acid is a strong free radical scavenger [57]. The smaller the IC_50_ value, the greater the antioxidant capacity of the samples [103]. Notably, CE/CsNPs exhibited the most potent antioxidant activity, with an IC_50_ value of 9.9 ± 0.12 µg/mL, comparable to ascorbic acid, as shown in Fig. 4B (*p* < 0.05). The CE results displayed moderate antioxidant activity with an IC_50_ value of 80.28 ± 1.2 µg/mL, while drug-free CsNPs showed weak antioxidant activity [11].

### *In vitro* anti-inflammatory and anticancer activities

**Fig. 5:**
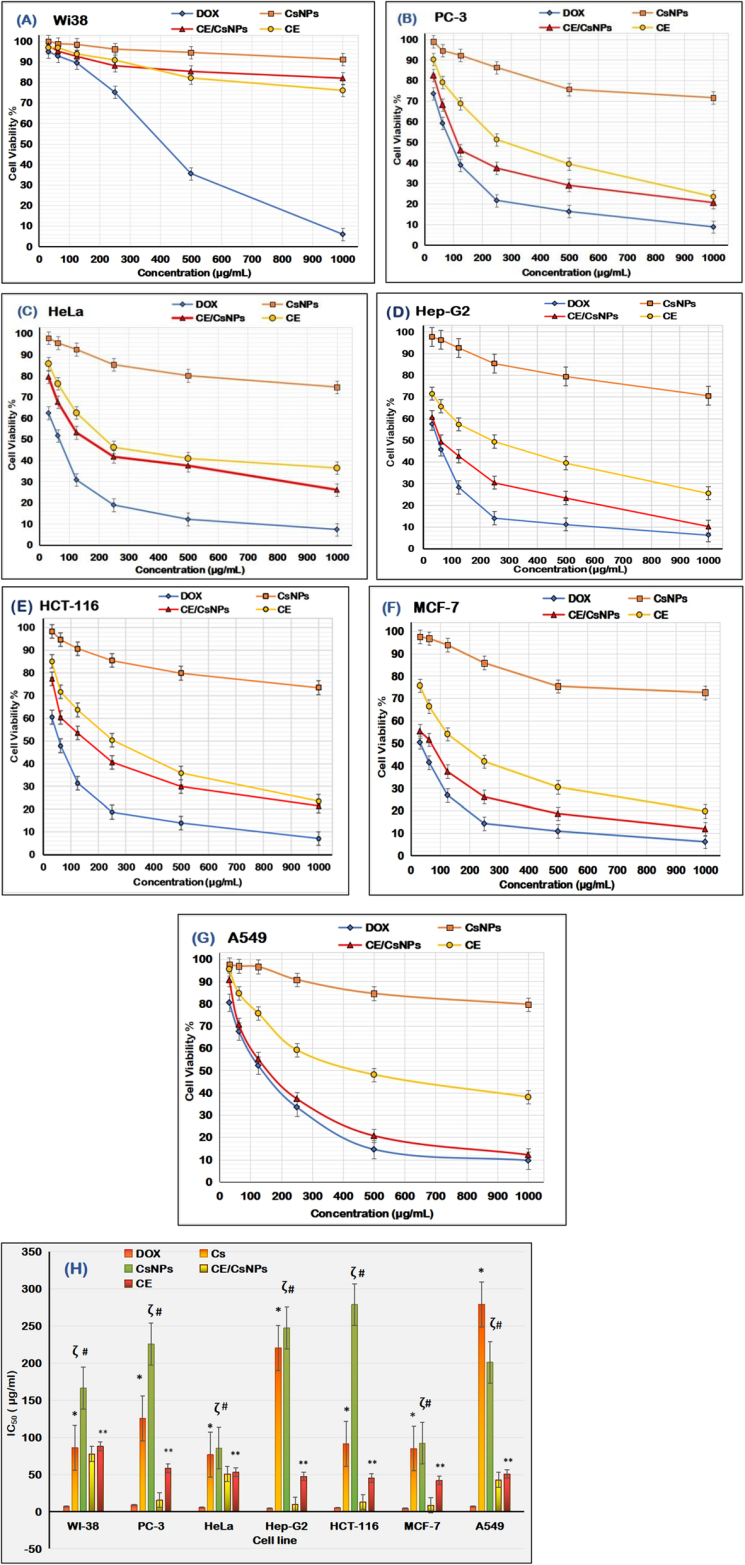
The anticancer effects of doxorubicin, CsNPs, CE/CsNPs, and CE solutions at different concentrations on tumor cell viability % of (**A**) normal fibroblast (WI38), **B** prostate cancer (PC3), **C** epitheliod carcinoma (HeLa), **D** liver cancer (Hep-G2), **E** colorectal cancer (HCT-116), **F** mammary gland (MCF-7), **G** lung cancer (A549) cells, and (**H**) half-maximal inhibitory concentration (IC_50_) values of DOX, Cs, CsNPs, CE/CsNPs, and CE solutions evaluated on different cancer cell lines in comparison to normal human cells (WI-38) 48 hr post-treatment (ζ *P* < 0.0001). *p < 0.05 CE/CsNPs *vs.* Cs group, ^#^ *p* < 0.05 CE/CsNPs *vs*. CsNPs group, ** *p* < 0.05 CE/CsNPs *vs.* CE. In all panels, *n* = 3, results are expressed as mean ± standard deviation. DOX: Doxorubicin, Cs: Chitosan powder, CE/CsNPs: Ethanolic extract *of C. comosum* loaded in chitosan nanoparticles, CE: Ethanolic extract of *C. comosum*, and CsNPs: Chitosan nanoparticles

Medicinal plants are a largely unexplored source of bioactive compounds for cancer therapy [91]. However, they have significant limitations, including nonspecific targeting, low water solubility, and limited therapeutic efficacy [14]. The study aimed to demonstrate the antitumor and anti-inflammatory capabilities of the synthesized CE/CsNPs and the free CE on different tumor cells. To evaluate the cytotoxic effects of the treatments, the results were compared with those of doxorubicin. Notably, CE/CsNPs showed the lowest toxicity to normal cells (WI-38), as illustrated in Fig. 5A, indicating their biocompatibility for targeted cancer therapy while reducing the adverse effects on healthy cells [8]. The MTT results revealed a significant dose-dependent anti-proliferative response against cancer cell lines; as the amount of employed compounds increased, the viability of the cancer cells decreased.

Undoubtedly, the CE/CsNPs possessed strong antitumor efficacy against different cancer cells, especially lung, breast, and liver cancers, with cell viability % ranging from 10.2 ± 0.05% to 60.7 ± 0.04% for Hep-G2 cells, 11.9 ± 0.05% to 55.4 ± 0.01% for MCF-2, and 9.11 ± 0.39% to 70.66 ± 0.74% for A546 cells. Treatment of PC-3, HeLa, and HCT-116 cells with CE/CsNPs showed moderate antitumor activity ranging from 20.6 ± 0.02% to 82.5 ± 0.05%, 26.1 ± 0.94% to 79.5 ± 0.45%, and 21.4 ± 0.74% to 77.4 ± 0.57%, respectively (Fig. 5B-G). The data related to the safety assessment of CE/CsNPs and CE was further confirmed through studying the IC_50_ of the treatments on cancer cells, as presented in Fig. 5H. Both treatments displayed decreased cytotoxicity against WI-38 cells by 11.5 and 13.2 folds in comparison to doxorubicin, with recorded values of 77.68 ± 1.32 µg/mL and 87.75 ± 2.15 µg/mL, respectively. In contrast, the cytotoxicity IC_50_ values after treatment of cancer cells with CE/CsNPs were 15.49 ± 0.35 µg/mL, 50.68 ± 1.54 µg/mL, 9.46 ± 1.12 µg/mL, 12.87 ± 0.27 µg/mL, 8.5 ± 0.17 µg/mL, and 42.6 ± 1.65 µg/mL for PC-3, HeLa, Hep-G2, HCT-116, MCF-7, and A549 cells, respectively, which indicated high to moderate cytotoxic activity on cancer cells. Thus, we can conclude that CE/CsNPs were more effective against lung, breast, and liver cancers than prostate, epithelioid, and colorectal cancers.

**Fig. 6:**
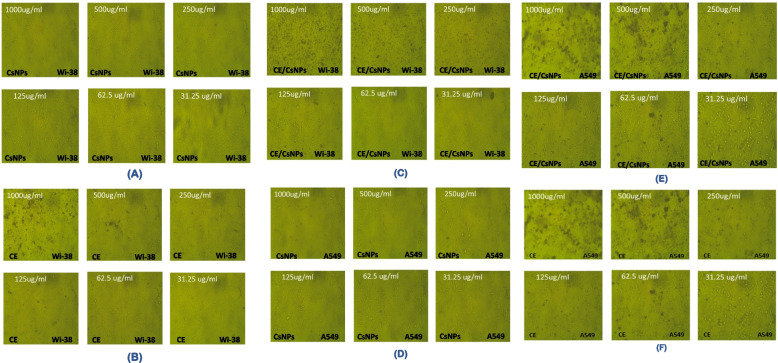
Photos of WI-38 (**A**, **B**, **C**) and -A549 (**D**, **E**, **F**) cells incubated with different concentrations (1000, 500, 250, 125, 62.5, and 31.25 μg/mL) of CsNPs, CE/CsNPs, and CE. CE/CsNPs: Ethanolic extract *of C. comosum* loaded in chitosan nanoparticles, CE: Ethanolic extract of *C. comosum*, CsNPs: chitosan nanoparticles, WI38: human lung fibroblast, and A549: human lung cancer cell lines

Furthermore, the microscopical examination of WI-38 and A546 cells was done to validate the cytotoxic effect of the tested compounds, the cells that were incubated with various concentrations (1000, 500, 250, 125, 62.5, and 31.25 μg/mL) of CsNPs, CE/CsNPs, or CE were analyzed (Fig. 6); the A546 cells treated with CE/CsNPs revealed large-scale morphological cytoplasmic vacuolization, which accompanies cell death, as illustrated in Fig. 6E [104]. The CE/CsNPs had a non-toxic protective effect against WI-38 at doses less than 125 μg/mL, whereas CE showed reduced cytotoxicity at concentrations less than 62.5 μg/mL (Fig. 6B & C).

**Fig. 7:**
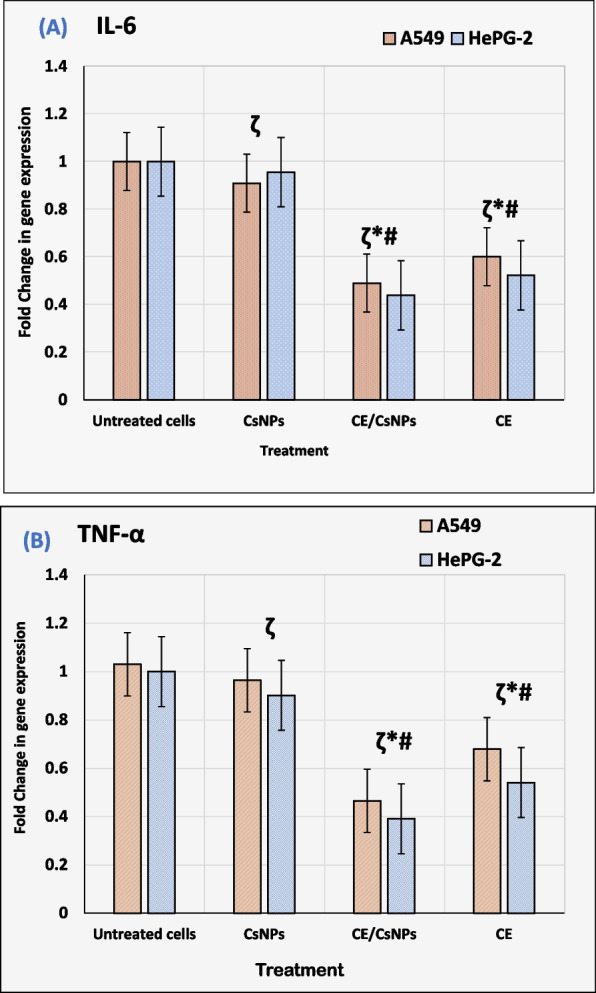
Relative expression of cytokines (**A**) interleukin 6 (IL-6) and (**B**) tumor necrosis factor α (TNFα) in A549 and Hep-G2 cell lysates 48 hr post-treatment with CsNPs, CE/CsNPs, or CE solutions at their corresponding IC_50_ values (ζ *P* < 0.0001). * *p* < 0.05 CE/CsNPs or CE groups *vs.* untreated group, # *p* < 0.05 CE/CsNPs *vs.* CE group. In all panels, *n* = 3, results are expressed as mean ± standard deviation. CE/CsNPs: Ethanolic extract of *C. comosum* loaded in chitosan nanoparticles, CE: Ethanolic extract of *C. comosum,* and CsNPs: chitosan nanoparticles

Regarding inflammation, RT-qPCR was conducted to study the relative expression of cytokines after 24 hr of treatment. The treatment of cells with CE/CsNPs successfully down-regulated the TNF-α expression by 54.9% and 54.1% in A549 and Hep-G2 cells, respectively. Furthermore, the expression of IL-6 was remarkably decreased by 50.9% and 47.7% in A549 cells and Hep-G2 cells, respectively, compared to the untreated cells (*p* < 0.05). Treating the cells with CE revealed down-expression of the TNF-α in A549 and Hep-G2 cells by 34.1% and 45.9%, however IL-6 expression was down-regulated by 39.8% and 47.1%, respectively, compared to the untreated cells (*P* < 0.05) (Fig. 7). Thus, the collected data is suggesting the mitigation of inflammatory response in the treated cells.

It could be speculated herein that the high anticancer and anti-inflammatory potencies of CE/CsNPs in comparison to free CE suitably could be related to the release of CsNPs loaded with CE directly into the cells, which will subsequently cause the nucleus to be directly affected and lead to the promotion of a high number of apoptotic genes, as argued by Alzahrani (2021) [8] and El-Naggar et al. (2024) [100]. Thus, the exposure of cancer cells to CE/CsNPs could consequently lead to the death of a massive number of cancer cells through apoptosis [86]. Furthermore, chitosan has a dissociation constant (pKa) of 6.5, which may help it become more protonated in the acidic environment of tumors than in normal cells; consequently, this leads to an enhanced anticancer activity against cancer cell lines [105].

In the current study, nineteen bioactive compounds with anticancer actions were detected in the plant ethanolic extract (CE) (Table 1). The rhoifolin, pregna-4,6-diene-3,20-dione (guggulsterone), and aspidofractinine that were detected at peak 25 are considered highly potent anticancer compounds that work through different mechanisms. A previous investigation displayed that rhoifolin successfully reduced the migration of breast cancer cells through suppressing the mitogen-activated protein kinases (MAPK) [95]. Moreover, guggulsterone could regulate the mTOR-AKT and Ras/NF-κβ proliferative signaling pathways, as reported by Gupta et al. (2023) [75]. Aspidofractinine is one of the indole alkaloids that has cytotoxic effects against various types of cancer cell lines [66]. Moreover, Abdullah and Omran (2024) [55] reported that geldanamycin and its analogs (peak 13) possess anticancer effects since they inhibit Hsp90 and protect against a variety of human malignancies. The 4'-Apo-*β*, *ψ* carotenoic methyl ester (peak 14) is a C₃₅ apo-carotenoid terpenoid compound known to inhibit breast tumor cell proliferation [57]. Cyclolanostan-3-ol (peak 20) is classified as a triterpene that was reported to possess antitumor activities and high cytotoxic effects against different cancer cell lines [62].

Considering the exhibited anti-inflammatory potencies of CE and CE/CsNPs, several bioactive compounds were also detected, such as stigmasterols, which belong to phytosterols that exist in various herbal plants that were detected in the CE as stigmasta-5,22-dien-3-ol (peak 24); concisely, their anti-inflammatory activity is mediated through inhibiting the production of cytokines and their ability to disrupt the TNF-α-VEGFR-2 axis, which will consequently suppress the migration and proliferation of endothelial cells [65]. Minigh (2007) [68] explained the anti-inflammatory and anti-proliferative properties of fluocinonide (peak 23), which is a potent corticosteroid. Impressively, fatty acids and their esters that were detected included Hexadecanoic acid, azelaic acid, eicosenoic acid, octanoic acid, sebacic acid, 10,13 eicosadienoic acid, and linoleic acid. These fatty acids possess anti-inflammatory potencies through the inhibition of cytokine production and repression of nuclear factor-κB (NF-κB) signaling [39, 42, 43, 47, 51]. These fatty acids are known for their anticancer effects [38, 45, 48, 50]. The presence of branched carbohydrates such as mannopyranose (peak 19) and glucopyranose (peak 20) can suppress the production of cytokines [69], stimulate the cell-mediated immune response, and promote immunomodulatory capabilities [63].

### Antimicrobial Effect of CE and CE/CsNPs

**Fig. 8:**
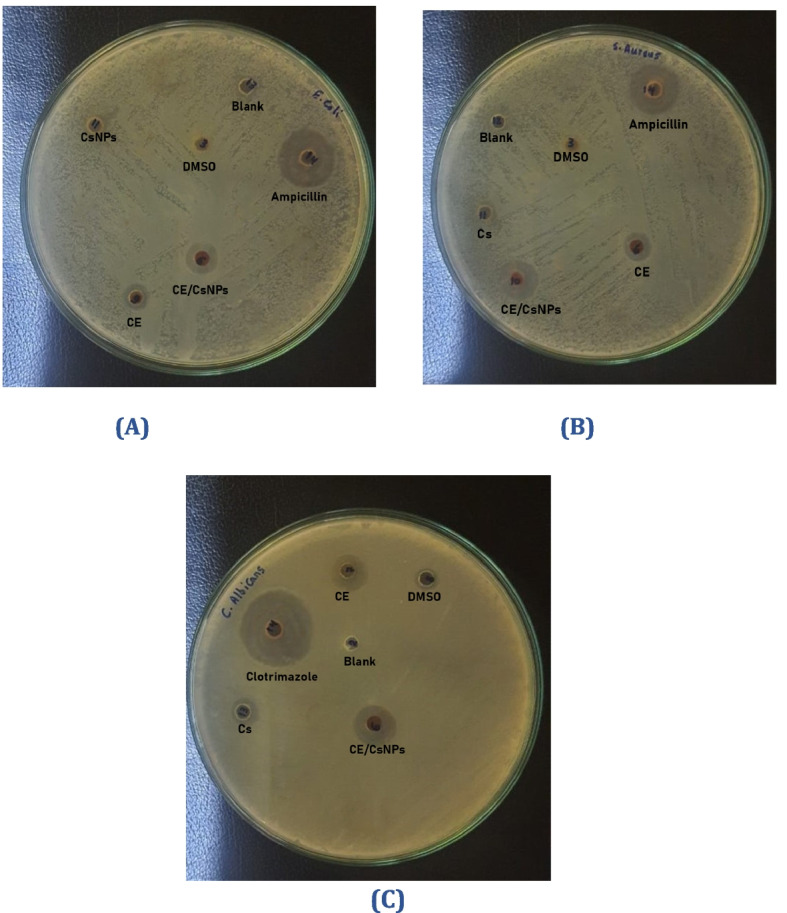
The antimicrobial activity of CsNPs, CE/CsNPs, and CE solutions against (**A**) *Escherichia coli* (*E. coli)*, **B** *Staphylococcus aureus* (*S. aureus),* and (**C**) *Candida albicans* (*C. albicans)*. DMSO: dimethyl sulfoxide, CE/CsNPs: Ethanolic extract *of C. comosum* loaded in chitosan nanoparticles, CE: Ethanolic extract of *C. comosum*, CsNPs: chitosan nanoparticles, and DMSO: dimethyl sulfoxide

The disc diffusion assay was performed to study the antimicrobial capabilities of Cs, CE, or CE/CsNPs [106]. The observations displayed that CE/CsNPs had the highest antibacterial effect among all treatments (*P* < 0.05) against pathogenic bacteria, including *E. coli* and *S. aureus* (Table 3 and Fig. 8A & B), with an inhibition zone of 12.34 ± 0.75 mm for *E. coli* and 16.41 ± 1.01 mm for *S. aureus*. Treatment with CE displayed an inhibition zone of 7.52 ± 0.80 mm and 10.54 ± 1.25 mm for *E. coli* and *S. aureus*, respectively. Additionally, the in vitro antifungal effects of CE/CsNPs were examined against *C. albicans* (Fig. 8C)*.* The treatment with CE/CsNPs showed more valuable results with an inhibition zone of 18.74 ± 1.13 mm compared to CE, which displayed an inhibition zone of 12.57 ± 1.54 mm.

**Table 3:** Antimicrobial effect of ethanolic extract of *C. comosum* (CE), chitosan nanoparticles (CsNPs), and CE-loaded chitosan nanoparticles (CE/CsNPs) showing the inhibition zone ± standard deviation, activity index, and percentage activity; Ampicillin and Clotrimazole are used as positive controls for bacteria and fungus, respectively

Treatment	*E. coli* gram-negative bacteria			*S. aureus* gram-positive bacteria			*C. albicans* fungus		
	**Diameter of inhibition zone (mm)**	**Activity Index (AI)**	**Percentage Activity (PA%)**	**Diameter of inhibition zone (mm)**	**Activity Index (AI)**	**Percentage Activity (PA%)**	**Diameter of inhibition zone (mm)**	**Activity Index (AI)**	**Percentage Activity (PA%)**
**CE**	7.58 ± 0.80^bc^	0.283	28.3	10.54 ± 1.25^bc^	0.438	43.8	12.57 ± 1.54^bc^	0.457	45.7
**CsNPs**	0.00 ± 0.0	0	0	0.00 ± 0.0	0	0	5.12 ± 0.95^abc^	0.186	18.6
**CE/CsNPs**	12.34 ± 0.75^ac^	0.464	46.4	16.41 ± 1.01^ac^	0.682	68.2	18.74 ± 1.13^ac^	0.683	68.3
**Ampicillin**	25.37 ± 0.03	1	100	23.54 ± 1.12	1	100.0	0.00 ± 0.0	0	0
**Clotrimazole**	0.00 ± 0.0	0	0	0.00 ± 0.0	0	0	28.41 ± 0.04	1	100.0

Data are expressed as mean ± standard deviation (*n* = 3). One-way ANOVA (*P* < 0.05), followed by a post-hoc test for comparison between groups, was used (^a^ *p* < 0.05 *vs* CE group, ^b^ *p* < 0.05 *vs* CE/CsNPs group, and ^c^ *p* < 0.05 *vs* control (Ampicillin for bacteria or Clotrimazole for fungi))

Interestingly, the application of CE/CsNPs to the examined strains resulted in the lowest MIC, MBC, and MFC values compared to all other treatments. The treatment of the fungal inoculum (*C. albicans*) with CE/CsNPs exhibited a significant MIC value of 15.62 μg/mL and MFC value of 31.25 μg/mL (Table 4). Moreover, the MIC and MBC concentrations for *S. aureus* were 15.62 μg/mL and 31.25 μg/mL, respectively, indicating that CE/CsNPs are more toxic to this microorganism compared to *E. coli*, which obtained MIC and MBC concentrations of 31.25 μg/mL and 62.50 μg/mL, respectively. The treatment of *S. aureus* with CE exhibited MIC and MBC values of 31.25 μg/mL and 125.00 μg/mL, respectively. While for *E. coli*, the treatment with CE displayed values of 62.5 μg/mL and 125.00 μg/mL, respectively. The MIC and MBC values of *C. albicans* were 31.25 μg/mL and 62.50 μg/mL, respectively, after treatment with CE. These results might be in good agreement with those investigations of Mohammed (2016) [107] and Alghamdi et al. (2023) [29], who observed the high antibacterial potential *of C. comosum*. Furthermore, exposure of *C. albicans*, *E. coli,* or *S. aureus* to CsNPs was associated with minimal antimicrobial activity, which was in accordance with Sobhani et al*.* (2017) [108].

**Table 4:** Minimal inhibitory concentration (MIC), minimum bactericidal concentration (MBC), and minimum fungicidal concentration (MFC) of ethanolic extract of *C. comosum* (CE), CE loaded Chitosan nanoparticles (CE/CsNPs), and chitosan nanoparticles (CsNPs) in μg/mL

Treatment	*E. coli* ATCC 8739		*S. aureus* (ATCC 6538)		*C. albicans* (ATCC 10221)	
	MIC (μg/ml)	MBC(μg/ml)	MIC(μg/ml)	MBC(μg/ml)	MIC(μg/ml)	MFC(μg/ml)
**CE**	62.50	125.00	31.25	125.00	31.25	62.50
**CE/CsNPs**	31.25	62.50	15.62	31.25	15.62	31.25
**CsNPs**	250.00	500.00	125.00	500.00	125.00	250.00

The observed differences in sensitivity between gram-positive and gram-negative bacteria after treatment are owing to the structural and compositional differences in their cell walls and the way they interact with the treatments [109, 110]. NPs were shown in studies to have effective antibacterial action via interfering with cell wall and membrane activities. This involves the generation of free radicals, which damage DNA, alter protein activities, and kill cells. The electrostatic interaction of negatively charged bacterial membranes with positively charged NPs has the potential to disrupt cellular integrity. Subsequently, this might cause harm to these structures, causing microbial death [111]. Drug-free CsNPs displayed weak fungicidal activity resulting from their high surface area, which permits them to adhere more strongly to fungal cell surfaces and impair their membranes'integrity [20]*.*

The CE contains esters of nonanedioic acid, hexadecanoic acid, and octadecanoic acid. These acids have antimicrobial properties through attenuating DNA/RNA replication, protein synthesis, and disrupting cell wall synthesis [81]. In addition, the extract contains cyclolanostan-3-ol [62] and furazan [72]. These compounds enhance the antibacterial effect through the glycosylation processes carried out by the phenolic components, which will then interact with the bacterial membrane structure [95]. A study by Bakrim et al. (2022) [70] explained that stigmasterol has antibacterial activity via its ability to change the bacterial membrane’s composition.

In the current results, the decrease in the MIC values of *E. coli* or *S. aureus* may be due to the capability of the NPs to improve the CE’s penetration into the bacterial cell, which in turn suppresses the bacterial growth. Seemingly, the preparation of CE/CsNPs displayed stronger antibacterial and antifungal capabilities in comparison to free CE. This reflects the potency of the synthesized NPs in improving the extracts’ bioactivities, along with the presence of citrate as a cross-linking agent, which possesses antimicrobial properties. A previous study illustrated that using NPs with a ZP near ± 30 mV improved microbial attack efficiency, which aligns with the findings of the present investigation [77]. According to El-Naggar et al*.* (2024) [100], the polycations of chitosan can interact with the bacteria's anionic surface proteins and lipopolysaccharides, altering the permeability barrier and inhibiting bacterial growth.

## Conclusion

The investigation highlights the efficiency of encapsulating ethanolic extract of Egyptian *C. cosmosum* (CE) in chitosan nanoparticles (CE/CsNPs) with eco-friendly properties by using citrate as a crosslinker for the in vitro assessment of its anticancer and antimicrobial activities. The CE possesses bioactive compounds with different biological capabilities. The synthesized CE/CsNPs-2, which was loaded with 350 mg/100 mL of CE, displayed the highest entrapment efficiency, so it was chosen to be studied further. The CE/CsNPs-2 displayed anticancer potency, which was reflected by the significant cytotoxic activity against cancer cells, as well as its low cytotoxicity to normal cells, suggesting its ability to be used in anticancer regimens for targeting cancer cells. The CE/CsNPs-2 exhibited antioxidant and anti-inflammatory activities, in addition to antimicrobial properties against gram-positive, gram-negative, and fungal strains, yielding more valuable results than treatment with the CE. This may pave the way for newly synthesized sustainable compounds to be candidates for novel natural anticancer and antimicrobial therapeutics.

## Supplementary Information

Supplementary Material 1

## Data Availability

No datasets were analyzed during the current study. Source data are available on request from the corresponding author. All other data needed to reproduce the results presented here can be found in the manuscript, tables and figures.

## Abbreviations

CE: Ethanol extract of *C*. *comosum*
Cs: Chitosan powder
CsNPs: Chitosan nanoparticles
CE/CsNPs: CE-loaded chitosan nanoparticles
EE: Entrapment efficiency
FT-IR: Fourier transform infrared spectroscopy
GC/MS: Gas chromatography mass spectrometry
IL-1β: Interleukin-1β
MBC: Minimum bactericidal concentration
MIC: Minimum inhibitory concentration
PS: Particle size
PDI: Polydispersity Index
TEM: Transmission electron microscopy
TNF-α: Tumor necrosis factor α
ZP: Zeta potential
