# Supplementary material for: Chitosan nanocarriers loaded with Egyptian Calligonum comosum L'Hér. Extract: an eco-friendly approach for investigating triple-action biological activities
Source: BMC Complement Med Ther. 2025 Sep 24;25:332. doi: 10.1186/s12906-025-05047-x (PMC12459056; doi:10.1186/s12906-025-05047-x)
Supplement: Supplementary file 1 — Supplementary Material 1 [file 12906_2025_5047_MOESM1_ESM.docx]

**Supplementary Materials For**

**“Chitosan Nanocarriers Loaded with Egyptian *Calligonum comosum*****L'Hér. Extract: An Eco-Friendly Approach for Investigating Triple-Action Biological Activities”**

Yasser I. Khedr^1^, Soliman M. Toto^2^, Salama M. El-Darier^2^, Mostafa K. Hafez^3^, Abdel-Hamid A. Sakr^1^, Magdi A. Ali^4^, Mohamed Zakaria El-Sayed^4^, Aya M. Helal^5^.

**This file includes:**

**Supplementary Figures 1-3**

**Supplementary Table 1**

**
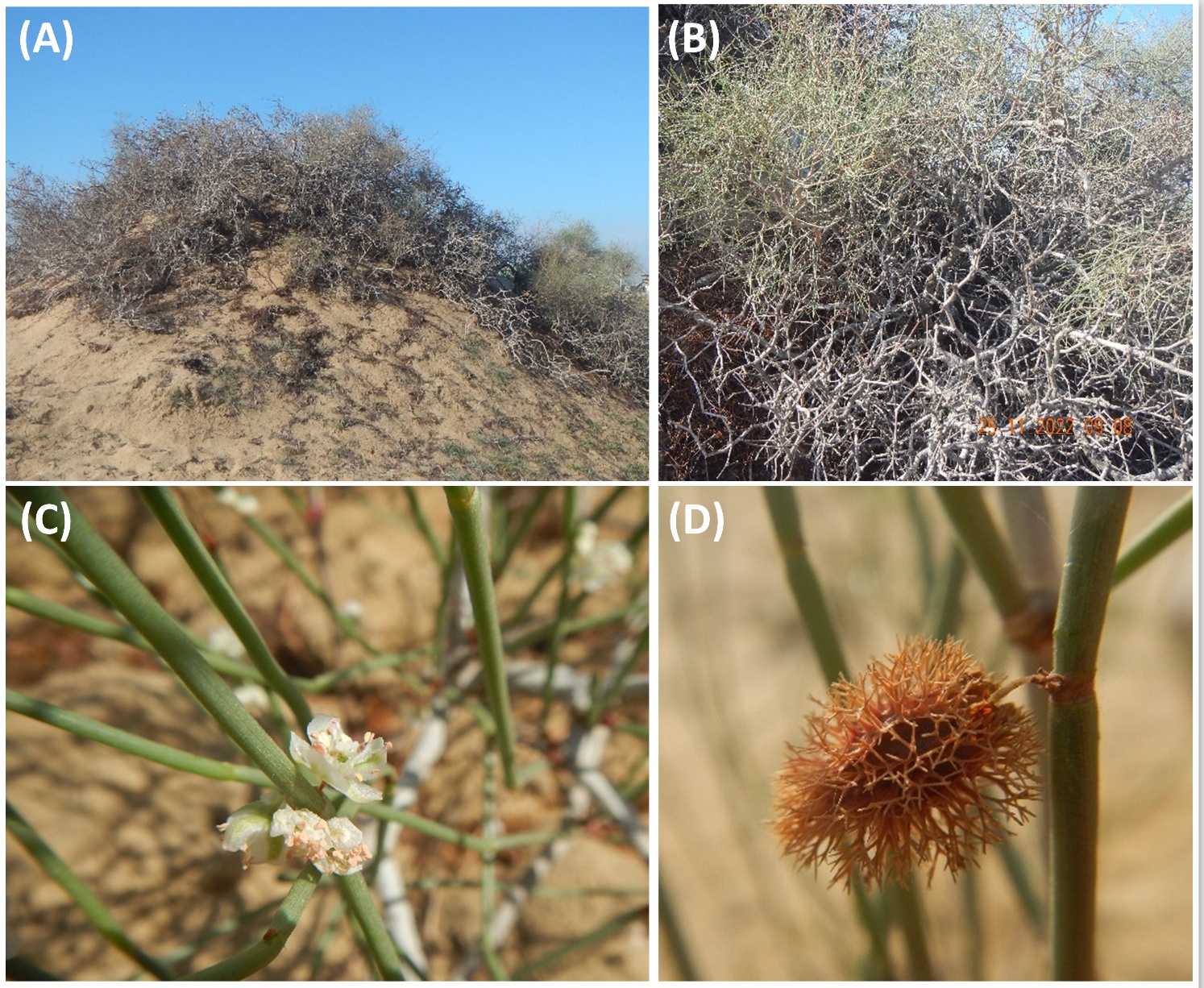
**

**Fig S1. Collected and identified Egyptian *C. comosum*** (A) Plant grown on a dune; (B) Branches of the plant; (C) Flowers, and (D) Fruit.


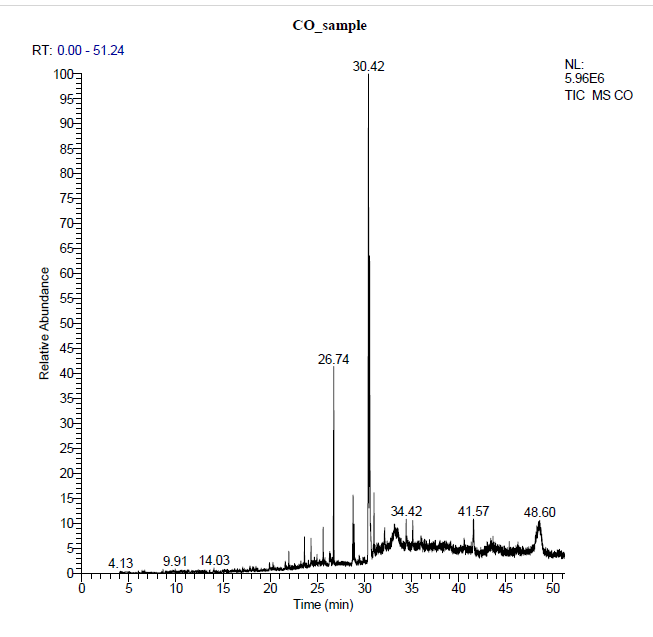


**Fig S2.** Gas chromatography-mass spectrometry profile of ethanolic extract of *C. comosum* (CE); RT: retention time.

**
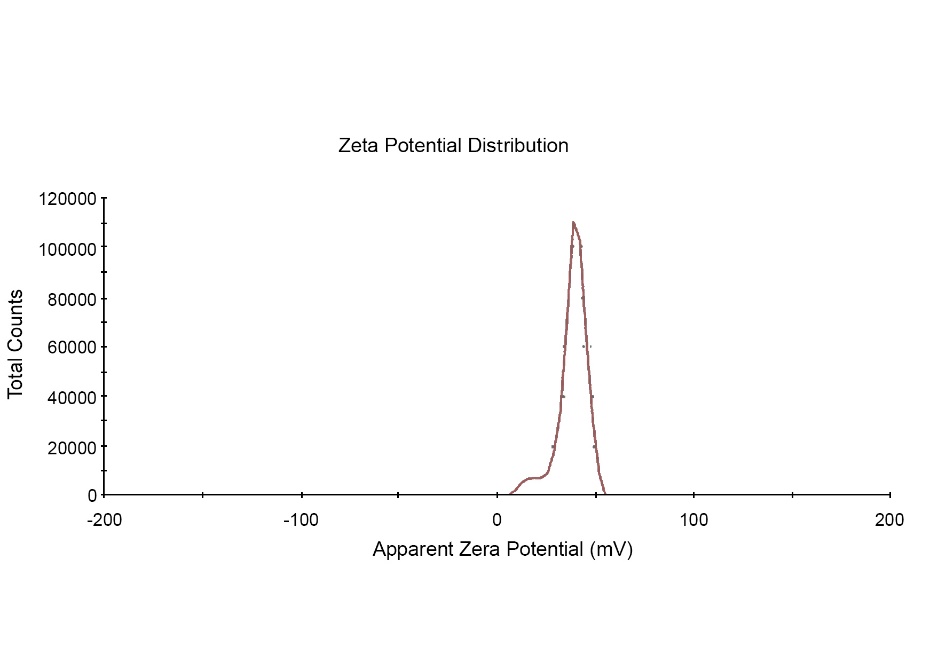

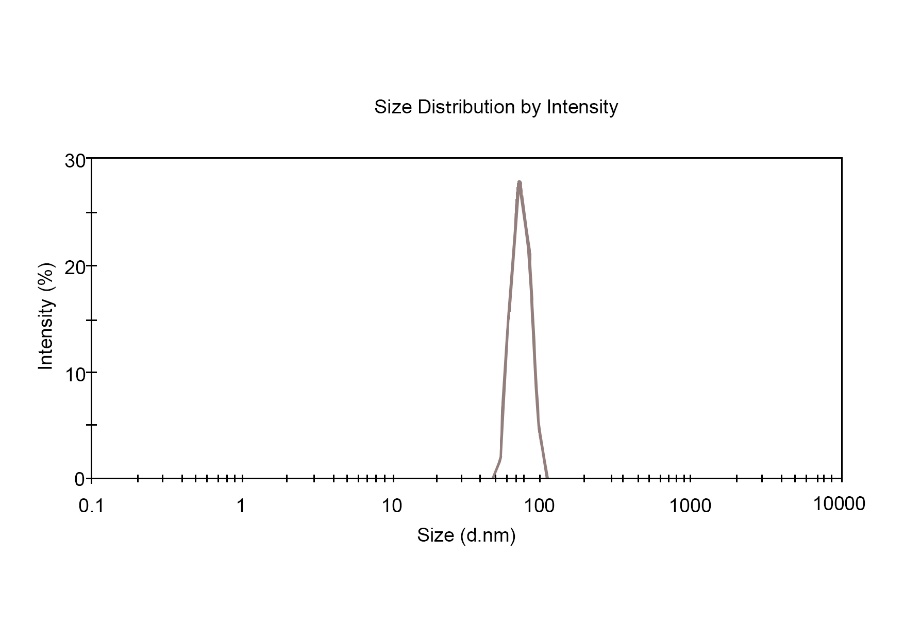
**

**(A)**

**(B)**

**Fig S3.** Dynamic light scattering for (A) particle size and (B) zeta-potential of CE/CsNPs-2 formulation.

**Supplementary Table 1. Primers used for RT-qPCR for the determination of proinflammatory cytokines**

| **Human gene** | **Sequence** |
| --- | --- |
| **IL-6** | **F:** TGATACGCCTGAGTGGCTGTCT |
|  | **R:** CACAAGAGCAGTGAGCGCTGAA |
| **TNF-α** | **F:** CAGCATAGAGCAGGACATGGAG |
|  | **R:** GAACAGCGGTAGTATCAGCCAG |
| **β-actin** | **F:** ATTGCTGACAGGATGCAGAAGG |
|  | **R:** TGCTGGAAGGTGGACAGTGAGG |
